# Reduced relational and item-specific processing in cognitive offloading

**DOI:** 10.1186/s41235-025-00647-0

**Published:** 2025-07-15

**Authors:** Hagit Magen, Michal Tomer-Offen

**Affiliations:** The Hebrew University of Jerusalem, Jerusalem, Israel

**Keywords:** Memory, Cognitive offloading, Distinctiveness theory, Relational processing, Item-specific processing

## Abstract

In many circumstances in everyday life, individuals offload information to external stores (e.g., shopping lists) to compensate for limitations in internal memory. When saving information externally, individuals tend to refrain from actively encoding an additional internal copy of the information, leading to a weakening of its internal trace. This study examined whether the loss of the internal trace due to offloading is limited to item-specific information (e.g., apples, milk) or extends to relational information as well (e.g., fruit, dairy products). In the first two blocks of each of two experiments, participants learned lists of 20 unrelated words, which they could save externally for use during a subsequent memory test. In the third block, participants learned a categorized list consisting of 6 exemplars from 8 semantic categories. While participants could save the list externally, they were prevented access to the list at test. Half of the participants were informed that the list would be unavailable at test, thus relying on internal memory, whereas the remaining participants trusted the list availability. Reliance on the external store led to a reduction in the internal trace of the offloaded information. Notably, saving the information externally resulted in decreased internal memory for both item-specific and relational information. This study indicates that internal memory for relational information does not effectively support the retrieval of information from external stores, and suggests that optimal organization of external stores should highlight relational information.

## Significance statement

The demands of everyday life often far exceed the capabilities of our internal memory. Consequently, individuals frequently offload information externally to aid daily tasks (e.g., writing shopping lists, setting reminders, saving information in computer files). Recent research has shown that when individuals use external stores, they tend to refrain from actively encoding the same information internally, leading to a weakening of the internal trace of the saved information. External stores hold information at various levels; shopping lists contain item-specific (e.g., apples, milk) and relational information shared among several items (e.g., fruit, dairy products), and computer files are stored in shared folders. The current study examined whether the loss of the internal trace due to offloading affects both item-specific and relational information. In the critical trial of each of the two experiments, participants learned a categorized list comprising six exemplars from eight different categories, which they then saved externally. The results showed that when participants relied on the external store to save the categorized list, they refrained from maintaining it internally, resulting in a loss of both item-specific and relational information. Thus, this study suggests that internal memory for relational information does not effectively support the retrieval of information from external stores. To improve performance and optimize the use of external stores, individuals should externalize relational information in addition to item information, integrating it into the organization of external stores.

## Introduction

The demands of everyday life often exceed the capabilities of our internal memory. Consequently, individuals frequently offload information to external stores to support daily tasks, such as writing shopping lists or setting reminders (Risko & Gilbert, 2016). The benefits of cognitive offloading are unequivocal, as a copy of the to-be-remembered information is created and is available to guide behavior (Eskritt & Ma, 2014; Gilbert et al., 2020; Grinschgl et al., 2021; Lu et al., 2020). Nevertheless, cognitive offloading has a detrimental effect on the internal trace of the offloaded information, supposedly similar to processes that occur when individuals intentionally remove items from memory in the item-method directed forgetting paradigm (Eskritt & Ma, 2014; Grinschgl et al., 2021; Kelly & Risko, 2019; Sparrow et al., 2011).

In the item-method directed forgetting paradigm, participants are presented with information that is denoted as to-be-remembered or to-be-forgotten for a future test. When memory for the to-be-forgotten items is compared to memory for the to-be-remembered items, a cost to the internal memory trace of the to-be-forgotten items is typically observed (Basden et al., 1993; Bjork, 1970; Fawcett et al., 2008; Macleod, 1998). A leading hypothesis posits that participants disengage from strategically encoding or maintaining the no longer relevant to-be-forgotten information, resulting in the observed reductions in memory performance in this condition (Basden et al., 1993; Bjork, 1970; Macleod, 1998; Tan et al., 2020). 

Similarly, it has been suggested that when individuals save information externally, they refrain from actively encoding and maintaining the same information internally, leading to the observed cost to the internal memory trace of the offloaded information (Eskritt & Ma, 2014; Kelly & Risko, 2019, 2022; Sparrow et al., 2011). The study-effort hypothesis assumes that when information is stored externally participants refrain from allocating mental effort that is associated with strategies that benefit internal memory (Kelly & Risko, 2022). This assumption is consistent with the widely accepted view that humans generally attempt to avoid effort, be it physical or mental, which is perceived as costly and aversive (Kool et al., 2010; Shenhav et al., 2021). When information is kept internally, the cost of engaging in active encoding strategies results in substantial benefits to memory accuracy (e.g., Unsworth et al., 2019). However, when high accuracy is guaranteed by the use of the external store in cognitive offloading, the cost associated with the implementation of encoding strategies is no longer required (i.e., the cost has no benefit), and participants forgo the use of these strategies, disregarding the potential cost to internal memory (Kelly & Risko, 2022).

External stores are used to support multiple tasks and contain information characterized by both unique item-specific details and shared relational information. For example, meetings (item-specific information) may share temporal or spatial characteristics (relational information), unique shopping list items can belong to categories like fruit or dairy, and computer files are typically organized within shared folders. Recent studies indicate that while saving information externally reduces memory for item-specific information, memory for relational information is preserved (Lu et al., 2020, 2022). This preservation of relational information potentially has important practical and theoretical implications. For instance, externally stored information is typically distributed across multiple locations (smartphones, computers, or notebooks), intact internal relational memory can facilitate access to the relevant locations and overall performance (Lu et al., 2020; Sparrow et al., 2011). Intact internal relational memory can also support the efficient retrieval of information from external stores. Internal memory for the day of scheduled meetings would lead individuals to consult their calendar on the day for specific details. Remembering the categories of items on a shopping list can assist individuals to navigate through the different areas of the store efficiently without having to constantly consult the list. Thus, intact memory for relational information can assist not only in locating the stored information but also in the efficient retrieval of information from the store itself, similar to the role relational information plays in facilitating retrieval from internal memory (Hunt, 2012). 

The findings that memory for relational information is spared in cognitive offloading contrasts with results from the item-method directed forgetting paradigm which demonstrates memory loss for both to-be-forgotten item-specific and relational information (Lee, 2008; MacLeod, 1975; Montagliani & Hockley, 2019). This discrepancy might highlight unique characteristics of each process and deepen our understanding of externalization effects. Given these practical and theoretical considerations, the current study examined the fate of item-specific and relational information in cognitive offloading through the theoretical framework of distinctiveness theory (Einstein & Hunt, 1980; Hunt, 2012).

The distinctiveness theory posits that two qualitatively different processes affect memory: processing of item-specific information and processing of relational information. Distinctive processing occurs when the unique aspects of encoded items are processed in the context of their shared characteristics (Einstein & Hunt, 1980, for reviews Hunt, 2012, 2013). Distinctive processing improves memory accuracy and reduces false memory relative to processing either type of information alone (Einstein & Hunt, 1980; Hunt, 2012; Hunt & Einstein, 1981). For example, processing items based on both relational information (e.g., fruit) and item-specific information (e.g., oval and yellow) provides more diagnostic information about the memorized item (banana), than either type alone.

Relational information and item-specific information serve distinct roles during memory retrieval (Einstein & Hunt, 1980; Hunt, 2012). Relational information organizes retrieval by facilitating a retrieval plan and defining search sets based, for example, on semantic categories. This organization enables items from each search set to be retrieved together, with item-specific information contributing by highlighting the unique aspects of each item within the set. Relational and item-specific processing support performance in free recall tests in which internally generated retrieval cues are necessary for successful retrieval. Recognition tests rely more on item-specific processing, which is instrumental for discriminating between targets and lures, particularly when they share the same semantic category (Einstein & Hunt, 1980; Hunt & Einstein, 1981; Hunt & Seta, 1984; Neely & Balota, 1981). Relational processing and the generation of relational retrieval cues are thought to be spontaneous, especially when the shared information is highly familiar, whereas item-specific processing and the generation of item-related retrieval cues are typically deliberate and mostly idiosyncratic (Hunt, 2012; Hunt & Seta, 1984; Hunt & Smith, 1996).

Two recent studies have examined the contribution of relational processing to memory performance in the context of cognitive offloading, using two conceptually similar tasks (Lu et al., 2020, 2022). Lu et al. (2020) used the Deese–Roediger–McDermott (DRM) paradigm (Deese, 1959; Roediger & McDermott, 1995), where participants learn a list of words that share a certain semantic theme (gist). This paradigm typically produces high rates of false recall of a single non-studied word that is strongly associated with the list’s theme. In a subsequent study, Lu et al. (2022) compared memory performance between categorized and unrelated word lists. Categorized lists typically yield superior memory performance due to the contribution of relational information during retrieval (e.g., Cofer et al., 1966; Saint-Aubin et al., 2005). False memory in the DRM paradigm and enhanced memory of categorized lists are both contingent on the intact processing of relational information. 

In both studies, participants saved the associated (Lu et al., 2020) or categorized and unrelated (Lu et al., 2022) lists of words they studied externally by writing them down or typing them into the computer. During a critical trial, participants saved the lists externally during study but were prevented access to the list during a subsequent free recall test. Half of the participants were informed that the list they created would not be available at test beforehand, and therefore memorized the study list internally (a condition referred to as the no-offloading condition). The remaining participants trusted that the list they created would be available at test and thus engaged in cognitive offloading (a condition referred to as the offloading condition).

The results of both studies revealed the typical reduction in internal memory accuracy in the offloading condition compared to the no-offloading condition. Nevertheless, the rate of false recall and the categorization effect were similar across conditions, indicating that relational processing persisted despite the offloading manipulation, and that the performance costs were limited to the loss of item-specific information. Lu et al., (2020, 2022) proposed that disengagement from active encoding processes in the offloading condition primarily affected the more demanding item-specific processing, leaving the spontaneous relational processing intact. They further suggested that list availability during study might have strengthened inter-item relations, even as item-specific processing diminished.

These findings from the cognitive offloading paradigm contrast with item-method directed forgetting results, which have shown impairment of both item-specific and relational information of the to-be-forgotten items. Studies have demonstrated lower false recall rates in the DRM paradigm and reduced categorization effects when words in the associated or categorized lists were denoted to-be-forgotten compared to lists consisting of to-be-remembered words (Lee, 2008; MacLeod, 1975; Montagliani & Hockley, 2019). These effects have been attributed to reduced elaborative encoding following a ‘forget’ cue, which weakened both item strength and associated themes or semantic categories, thereby limiting the formation of inter-item associations (Montagliani & Hockley, 2019).

This apparent contradiction between the cognitive offloading and the item-method directed forgetting paradigms may be resolved by considering the distinct demands of each task. As indicated above, Lu et al (2020) suggested that exposure to the list during study might strengthen inter-item relations, even if processing of the items themselves was reduced. The impact of the list availability on relational processing would have been particularly robust in Lu et al.’s studies, which used lists of 10–15 words that were associated with a single theme or a single semantic category. In comparison, the item-method directed forgetting studies showing reduced relational processing typically employed longer multi-category lists with less robust relational information (e.g., Montagliani & Hockley, 2019). Therefore, the preservation of relational information in cognitive offloading might depend more on the robustness of inter-item associations than on list availability during study.

## The current study

The current study investigated the effects of cognitive offloading on item-specific and relational information processing. Following Risko and colleagues’ methodology (e.g., Kelly & Risko, 2019; Lu et al., 2020, 2022), the first two blocks of the experiment were designed to establish participants’ trust in the external store. Participants learned lists of 20 unrelated words that they saved externally by typing them into the computer, with access permitted during recall. In the critical third block, participants learned a categorized list comprising six exemplars from eight semantic categories. All the participants saved the list externally and did not have access to the list during a subsequent free recall test. Participants were either informed that the list would be unavailable at test and therefore memorized the study list internally (the no-offloading condition), or they trusted that the list would be available at test, similar to the first two blocks (the offloading condition). Memory was assessed in a free recall and a recognition test, the latter providing additional measures of item-specific processing (Hunt & Einstein, 1981).

We employed direct measures of both relational and item-specific processing to examine the part they took in retrieval from internal memory following cognitive offloading. Relational processing was evaluated by measures of category access (CA) and the adjusted ratio of clustering (ARC) (Einstein & Hunt, 1980; Hunt & Seta, 1984). CA reflects the number of categories that are retrieved during a free recall test (a category is accessed if at least one exemplar is retrieved from the category). It is assumed that when an item is retrieved, relational information pertaining to the item’s semantic category was accessible during retrieval. Thus, the CA score indicates the degree to which semantic categories were utilized as relational cues during retrieval (Hunt & Seta, 1984; Tulving & Pearlstone, 1966). The ARC measures the level of categorical clustering during retrieval (i.e., the tendency of items from the same category to be recalled together), and it adjusts for recall differences across experimental conditions, as would be expected in the comparison of the no-offloading and offloading conditions (Einstein & Hunt, 1980; Roenker et al., 1971). Increases in both the CA and the ARC have been associated with experimental manipulations that enhance relational processing (e.g., Einstein & Hunt, 1980; Hunt & Einstein, 1981; Hunt & Seta, 1984).

Item-specific processing was estimated from the free recall test using the items per category accessed (IPCA) measure. Once a category has been accessed during retrieval, item-specific retrieval cues provide the details enabling the retrieval of additional category exemplars (Hunt & Seta, 1984). The IPCA is therefore associated with the availability of item-specific information. The IPCA reflects the number of items that were recalled only from accessed categories, thereby adjusting for recall differences across experimental conditions. Additional measures of item-specific processing of hits and false alarm rates were obtained from the recognition test. Item-specific information facilitates the discrimination between targets and lures in recognition tests, especially when discrimination cannot be based on relational information (Einstein & Hunt, 1980). Enhanced item-specific processing has been associated with increases in the IPCA and recognition scores (e.g., Einstein & Hunt, 1980; Hunt & Einstein, 1981; Hunt & Seta, 1984).

Based on previous research, we predicted that cognitive offloading will negatively impact overall memory performance in both recall and recognition tests due to reduced item-specific processing. Given our use of less robust relational information compared to previous studies, we expected to observe reduced relational processing, consistent with findings from the directed forgetting literature. Overall, this study aims to clarify the differential effects of cognitive offloading on distinct types of memory processing.

## Experiment 1

### Methods

#### Participants

Forty students participated in the study (23 female, *M*_*age*_ = 24.78, *SD* = 2.86) for course credit or payment, all the participants were native Hebrew speakers. Sample size was calculated using G Power (Faul et al., 2007), to detect a medium-large sized effect in overall accuracy (*d* = 0.8, *α* = 0.05, power of 0.80, one-tailed), when comparing memory performance of participants in the offloading and no-offloading conditions. The calculation was based on large size effects that were detected in a previous study (Lu et al., 2020), and on pilot data from our laboratory. The study was approved by the Hebrew University Ethics Committee. The study was conducted online using the Zoom software, all the participants signed an electronic informed consent form before participating in the study. Participants provided an additional verbal consent at the beginning of the Zoom meeting.

#### Apparatus and stimuli

Experiment 1 was conducted online using Zoom. Participants used their personal computer, which was equipped with a camera and speakers. During the task, participants divided their screen between the Zoom window which was used by the experimenter to present the to-be-remembered lists of words, and a text file that was shared with the participants (i.e., a Google document), and was used to type in the lists of words during the study and test phases. The participant decided where to place the Zoom and the text file windows across the screen.

The stimuli consisted of three lists of words, which were presented sequentially at the center of the Zoom window (see Appendix). The first two lists included 20 unrelated words and were used to familiar the participants with the task and increase their trust in the external store. Lists of unrelated words were used to maintain participants’ attention on the list as an external store without biasing performance toward relational processing. The third critical list consisted of 6 exemplars from 8 semantic categories, for a total of 48 words. The words from the different categories were presented in random order. Additional 48 words (6 additional exemplars from the same 8 semantic categories) were used as distractors in the recognition test. Across participants, each list appeared equally often as the target or distractor list.

#### Procedure

At the beginning of the experiment, participants worked through a short practice block, which included a short list of 5 unrelated words (using the same method as in blocks 1–2), followed by the 3 experimental blocks. Each block consisted of a study phase, a 30-s delay phase and a test phase. At the beginning of the experiment, the experimenter shared their screen with the participants, and at the beginning of each block the experimenter shared with the participants links to two Google documents that were used by the participants to type in the lists of words in the study and the test phases.

*Blocks 1–2. Study phase.* Each word in the list was presented in the middle of the Zoom window for 5 s. Participants typed each word in the Google document that was shared with them. At the end of the study phase, the participants were asked to close the document, and the experimenter verified that the document was no longer available to the participant.

*Delay Phase*. A 30-s delay was introduced between study and test, during which the participants counted backwards by 3s. The experimenter monitored the participants performance.

*Test Phase*. At the end of the delay phase, the participants were asked to open the second document that was shared with them at the beginning of the block. They were asked to retrieve the words from the study list using the list that they created, which was presented by the experimenter on the Zoom window. The participants closed the document at the end of the test phase.

*Block 3. Study phase.* The study phase in this block was similar to the first two blocks, except that half of the participants, who were assigned to the no-offloading condition, were informed that the list that they would type in during the study phase would not be available at test. The remaining participants, who were assigned to the offloading condition, were unaware of this manipulation. All the participants were informed that the third block would be longer than the previous blocks, but were not informed of the categorized nature of the list.

*Delay Phase.* The delay phase was the same as in the first two blocks.

*Test Phase*. The test phase began with a free recall test. All the participants were asked to retrieve the words from the study list relying only on their internal memory. As in the previous blocks, the participants typed the words into the second Google document that was shared with them at the beginning of the block. Participants were given 150 s to retrieve the words, after which they were asked to close the text document.

Following the free recall test, the participants completed an additional recognition test. Participants were presented with a list of 96 words (presented in a random order) consisting of 48 words from the study list and 48 novel words that served as distractors. Each word was presented for 5 s on the Zoom window, and participants provided verbal yes/no responses, responding ‘yes’ if the word had appeared in the study list, and ‘no’ if the word was novel. The responses were coded by the experimenter. The experiment lasted approximately 30 min.

The data of Experiment 1 can be found on the Open Science Framework, at the link https://osf.io/7mrv4/?view_only=b05f70afd1a545b3a055ed1848dbae7a.

### Results and discussion

#### Blocks 1–2

In the first two blocks, the participants successfully used the lists they created during the study phase to retrieve the words. Participants retrieved on average 19.98 and 20 words from the first and second blocks, respectively.

#### Block 3

The data from the memory test in the third block were analyzed to determine the impact of the offloading manipulation on overall memory performance, and on relational and item-specific processing. Preliminary analyses showed that list order (i.e., which categorized list was the target list and which was the distractor list) had no impact on the results, and therefore, the data were collapsed across the two lists. All the analyses were based on independent-samples *t* tests, comparing the performance of participants in the offloading and no-offloading conditions. The data were analyzed using IBM SPSS Statistics (version 29).

In each group, participants committed intrusion errors, retrieving words that were not presented in Block 3. Because of the small number of cases, we did not analyze this data. On average participants in the offloading condition retrieved 2.60 words, whereas the participants in the no-offloading condition retrieved 1.15 words. The type of intrusion errors was similar in the two groups divided approximately equally between words from previous blocks (0.45 and 0.85 in the no-offloading and offloading conditions), novel words from the presented categories or category names (0.35 and 1.05 in the no-offloading and offloading conditions) and unrelated words (0.35 and 0.70 in the no-offloading and offloading conditions).

**Fig. 1:**
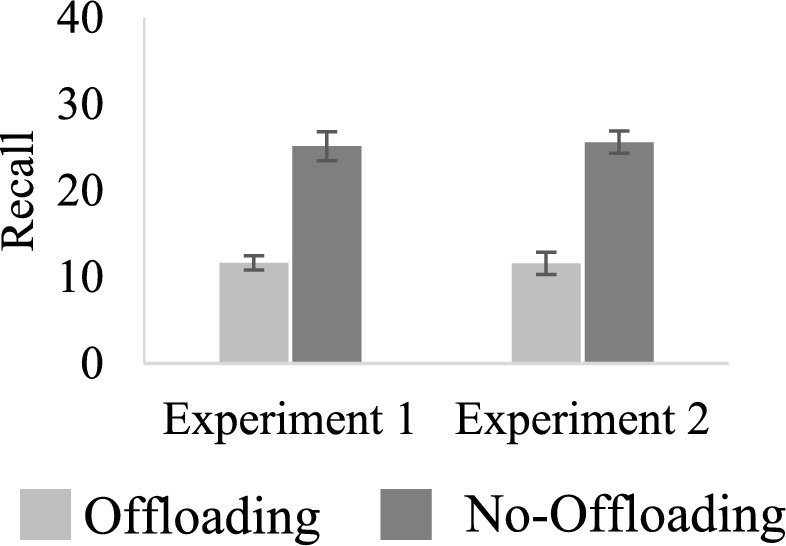
The number of words retrieved in the two offloading conditions in experiments 1 and 2. *Note*. Error bars represent standard error of the mean

The results of the free recall test are depicted in Fig. 1. Replicating previous observations, the number of words that the participants recalled was significantly higher in the group of participants who relied on internal memory (i.e., in the no-offloading condition), compared to the group of participants who trusted that the list they created would be available at test (i.e., in the offloading condition) *t*(38) = 7.22, *p* < 0.001, Cohen’s *d* = 2.28.

**Fig. 2:**
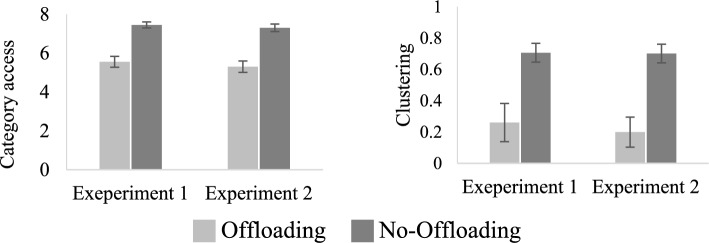
Category access (CA) (left) and mean clustering (ARC) (right) in the two offloading conditions in experiments 1 and 2. *Note*: Error bars represent standard error of the mean

*Measures of Relational Processing*. Participants in the offloading condition showed reductions in both measures of relational processing (see Fig. 2). The CA measure was significantly higher in the no-offloading condition compared to the offloading condition *t*(38) = 5.86, *p* < 0.001, Cohen’s *d* = 1.85, as was the ARC measure *t*(38) = 3.28, *p* < 0.01, Cohen’s *d* = 1.04. These results demonstrated that maintaining information externally significantly reduced the number of categories that participants retrieved and the level of clustering, reflecting the tendency of items from the same category to be retrieved together.

**Fig. 3:**
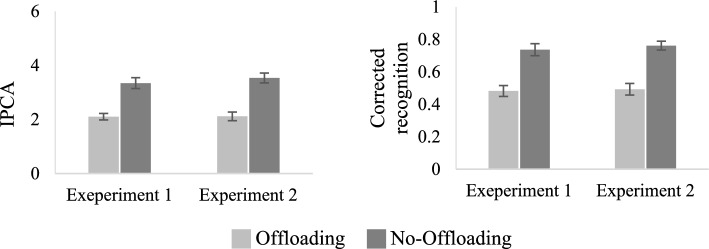
Mean IPCA (Left) and corrected recognition rate (hits–false alarms)(right) in the two offloading conditions in experiments 1 and 2. *Note*. Error bars represent standard error of the mean

*Measures of Item-specific processing*. The first measure of item-specific processing, the IPCA was derived from the free recall test. As depicted in Fig. 3, participants who relied on internal memory retrieved a larger number of items from each accessed category compared to the participants who trusted the external store *t*(38) = 5.33, *p* < 0.001, Cohen’s *d* = 1.69. Additional measures of item-specific processing were obtained from the recognition test. As shown in Table 1, the no-offloading condition yielded higher hit rates and lower false alarm rates. The corrected recognition score (hit minus false alarm rates) (Fig. 3) was significantly higher in the no-offloading condition *t*(38) = 5.07, *p* < 0.001, Cohen’s *d* = 1.60. Additional analyses demonstrated significant differences between groups in both hit rates *t*(38) = 5.04, *p* < 0.001, Cohen’s *d* = 1.59 and false alarm rates *t*(38) = − 2.61, *p* < 0.05, Cohen’s *d* = − 0.82.

**Table 1:** Means (SD) of hit and false alarm rates for the offloading and no-offloading conditions in experiments 1 and 2

	Experiment 1		Experiment 2	
	Hits	False alarms	Hits	False alarms
No-offloading	.85 (.11)	.11 (.10)	.86 (.09)	.09 (.09)
Offloading	.68 (.10)	.20 (.11)	.68 (.12)	.19 (.13)

The results of Experiment 1 replicated prior research demonstrating that storing information externally results in internal forgetting of the stored information. Contrary to previous findings, the results revealed reduced processing of item-specific and relational information. The CA and especially the ARC measures showed that participants who maintained items externally relied less on relational cues during retrieval compared to the participants who relied on internal memory, as they tended to retrieve items from the same semantic category in isolation rather than in clusters.

Our task design deviated from Lu et al. (2022) in several key aspects. Specifically, in this study, participants were presented with lists of unrelated words in the first two blocks. Additionally, the third block was relatively long, and the category exemplars in this block were presented in random order. Despite these differences, the relatively high CA and ARC observed in the no-offloading condition suggested that none of these manipulations prevented participants from detecting the categorized nature of the list or from using the relational information to support retrieval. Therefore, this finding supports our conclusion that the reduced relational processing observed in the offloading condition was due to the offloading manipulation itself rather than the specific task characteristics.

Furthermore, the results indicated that reliance on external storage resulted in internal cost to both recall and recognition, with reduced hit rate and increased false alarm rate. These findings are consistent with a recent study showing a cost in internal memory in tests of recognition in cognitive offloading (Kelly et al., 2025).

## Experiment 2

Experiment 2 was a close replication of Experiment 1 conducted in-person in the laboratory. The main goal of the experiment was to verify the results of Experiment 1 related to the impact of cognitive offloading on relational and item-specific processing.

### Method

The method of Experiment 2 was similar to Experiment 1 except for the following.

#### Participants

Similar to Experiment 1, 40 students from the same pool as in Experiment 1 participated in this study (35 female, *M*_*age*_ = 24.15, *SD* = 2.90). The study was approved by the University Ethics Committee, and all the participants provided written informed consent before participating in the study for course credit or payment.

#### Apparatus and stimuli

Participants performed the task in the laboratory in front of a desktop computer. As in Experiment 1, the screen was split in half. The target words were presented on one half of the screen, and a local text file was presented on the other half. Participants typed in the lists of words in the study and test phases into the local text file. The same stimuli were used as in Experiment 1.

The data of Experiment 2 can be found on the Open Science Framework, at the link https://osf.io/7mrv4/?view_only=b05f70afd1a545b3a055ed1848dbae7a.

#### Procedure

The procedure was the same as in Experiment 1. The experiment lasted approximately 30 min.

### Results and discussion

The analysis was the same as in Experiment 1.

#### Blocks 1–2

Participants successfully used the lists they created to retrieve the words at test. Participants retrieved on average 19.93 and 19.95 words in the first and the second blocks of the experiment, respectively.

#### Block 3

In each group, participants committed intrusion errors, retrieving words that were not presented in Block 3. In the no-offloading group, participants erroneously retrieved on average 0.2 words all semantically related words. In the offloading group 0.85 words on average appeared in previous blocks, 0.35 words were novel semantically related words, and 0.15 words were novel unrelated words.

The results of the free recall test are depicted in Fig. 1. Similar to Experiment 1, recall was higher in the no-offloading condition compared to the offloading condition *t*(38) = 7.73, *p* < 0.001, Cohen’s *d* = 2.44.

*Measures of relational processing*. The CA and the ARC were significantly larger in the group of participants who were assigned to the no-offloading condition *t*(38) = 5.72, *p* < 0.001, Cohen’s *d* = 1.81, and *t*(38) = 4.44, *p* < 0.001, Cohen’s *d* = 1.40, respectively (see Fig. 2). Similar to Experiment 1, storing information externally reduced the amount of relational information that was saved internally and consequently the use of relational cues to guide retrieval.

*Measures of item-specific processing*. Similar to Experiment 1 (see Fig. 3), the IPCA was significantly higher in the no-offloading condition compared to the offloading condition *t*(38) = 5.84, *p* < 0.001, Cohen’s *d* = 1.85, as was the corrected recognition rate *t*(38) = 5.93, *p* < 0.001, Cohen’s *d* = 1.88 (Fig. 3). As shown in Table 1, compared to the offloading condition, the no-offloading condition yielded significantly higher hit rates *t*(38) = 5.40, *p* < 0.001, Cohen’s *d* = 1.71, and significantly lower false alarm rates, *t*(38) = − 2.63, *p* < 0.001, Cohen’s *d* = − 0.83.

Overall, the results of Experiment 2 replicated those of Experiment 1 demonstrating reduced relational and item-specific processing in cognitive offloading.

## General discussion

The present study investigated whether the loss to the internal trace of information stored externally is limited to item-specific information or extends to relational information as well. In the critical final block of each of two experiments, participants saved lists of 48 words into the computer, consisting of 6 exemplars from 8 semantic categories. Memory was tested without access to the list, using free recall and recognition tests. Half of the participants were informed about the unavailability of the list during test, while the other half expected access to the list. The results of the two experiments revealed the typical loss of internal memory traces for externally stored information, as participants who relied on the list remembered fewer words compared to the participants who encoded the words internally. A detailed analysis revealed that external storage impaired both item-specific and relational processing. Participants who trusted the external store not only retrieved fewer items per category and showed lower hit rates with higher false alarms (indicating reduced item-specific processing), but also demonstrated diminished relational processing through reduced category clustering during retrieval and access to fewer categories overall.

Across the two experiments, the results indicate that the loss of the internal trace of externalized information stems from decreased distinctive processing (Hunt, 2012). The impaired performance reflects reduced contribution of both relational and item-specific cues to retrieval. These findings align with the hypothesis that individuals refrain from actively encoding items that they save in external stores (e.g., Kelly & Risko, 2022). This disengagement diminishes the processing of item characteristics, including their semantic category association, which subsequently interferes with the formation of inter-item relations similar to the effects observed in studies using the directed forgetting paradigm (Montagliani & Hockley, 2019).

These findings contrast with previous observations suggesting that relational information is preserved in cognitive offloading (Lu et al., 2020, 2022). One potential explanation for the discrepant results might be related to the different measures used to evaluate the contribution of relational information to performance. Lu et al. (2022) compared memory performance for categorized versus unrelated lists, whereas our study provided a more detailed analysis of the number of retrieved categories and the extent to which relational information guided retrieval. While both approaches rely on intact processing of relational information (Einstein & Hunt, 1980; Hunt, 2012), they may nevertheless depend on different aspects or differential strength of the relational information. Thus, relational information may persist or diminish in cognitive offloading depending on the manipulations and measures used to probe its contribution to memory performance. Examining this issue would be an interesting topic for future studies.

A second explanation for the discrepant results relates to the robustness of the relational information across studies. While Lu et al., (2020, 2022) used shorter lists of 10–15 words from a single semantic category or theme, this study employed a more complex list of 48 words across 8 semantic categories that were presented in a random order, reducing the strength of the relational information. Indeed, previous research has shown that presenting category exemplars in random order compared to a blocked presentation reduced clustering and overall recall (Cofer et al., 1966). Additional studies have demonstrated that the number of semantic categories and category exemplars in a list influences the strength of relational information and its impact on memory performance. For example, when list length was held constant, lists with fewer categories (and hence more exemplars in each category) were associated with better recall (Tulving & Pearlstone, 1966). Additionally, category size (i.e., the relative number of items representing each category) affects relational and item-specific processing (Hunt, 2012; Hunt & Seta, 1984). Large categories (12 exemplars or more) promote relational processing by focusing attention on the relational information, while small categories (fewer than 4 exemplars) encourage item-specific processing.

Furthermore, false recall rates in the DRM paradigm increase with the number of associated words, likely due to the formation of a stronger gist representation in longer single theme lists (Robinson & Roediger, 1997). Thus, the lists used in Lu et al., (2020, 2022) may have fostered more durable relational representations that persisted even when information was saved externally. However, such lists may not adequately represent real-world scenarios involving more complex information structures comprising of less robust and more elaborate relational information.

This explanation may also reconcile seemingly contradictory findings between cognitive offloading studies and item-method directed forgetting research, which has shown reduced processing of both item and relational information (e.g., Lee, 2008; Lu et al., 2020, 2022; March et al., 2005; Montagliani & Hockley, 2019). Similar to the current study, previous directed forgetting studies employed long lists with multiple themes or semantic categories (Lee, 2008; March et al., 2005; Montagliani & Hockley, 2019), supporting the assumed parallels between cognitive offloading and directed forgetting. Exposure to the written list during the study phase in cognitive offloading tasks does not appear to contribute to the formation of inter-item relations given the reduced processing of the items themselves.

Relational cues play a crucial role in internal memory retrieval (Hunt, 2012). The current results indicate that their role in supporting retrieval from external stores is more limited than previously suggested (Lu et al., 2022; Sparrow et al., 2011). For example, individuals often save information across multiple locations, such as different devices (smartphones, computers, or notebooks) or within various folders on a single device, forming a transactive memory system (Sparrow et al., 2011; Wegner, 1987; 1995). Earlier research proposed that internal memory for the location of stored information (‘where’) could substitute internal memory for the content itself (‘what’) (Lu et al., 2022; Skulmowski, 2023; Sparrow & Chatman, 2013); this would allow individuals to easily locate the externally stored information, even if the information is stored across multiple locations, such as computer folders. However, the current findings indicate that even this locational relational information may suffer degradation. In practical terms, if individuals lose access to their external stores (e.g., misplacing a shopping list or a calendar), their memory for organizational categories (product types or days of the week in which meetings take place) would be compromised, limiting their ability to support the reconstruction of the lost information. These findings further stress the importance of maintaining external access.

Nevertheless, the implications of this study extend beyond mere information recovery from internal memory, as using relational cues to retrieve information when access to the external store is granted represents an important aspect of retrieval in cognitive offloading. For instance, efficient use of shopping lists involves minimizing time spent in stores while maximizing the number of purchased items (Block & Morwitz, 1999). Internal memory of product categories could potentially guide store navigation without constant reference to the list, reducing shopping time and improving task efficiency, yet our results suggest this benefit may be limited. Thus, the broader implications of the current study emphasize the need to develop strategies that would optimize retrieval from external stores, whether when locating information across multiple devices or completing everyday tasks such as grocery shopping.

The extensive use of external stores for multiple everyday tasks brings new challenges in terms of retrieval. Schonpflug (1986) suggested that individuals must possess an internal representation of the overall structure of external stores and knowledge of their functional characteristics to allow for successful retrieval. This might involve intentional organization of external stores during their construction to facilitate performance (e.g., systemic computer folder structure) and incorporation of explicit relational information into the external store (e.g., organizing shopping lists by categories). Implementing structure and organization into external stores would require the allocation of cognitive resources during encoding.

The willingness of individuals to implement such organization remains uncertain, given their tendency to disengage from active encoding processes when using external stores due to study-effort considerations (Kelly & Risko, 2022; Kool et al., 2010). However, research thus far has indicated that individuals refrain from allocating mental effort to processes that facilitate internal memory performance which are less instrumental when information is saved externally. Future research should investigate whether disengagement from active encoding is limited to strategies that benefit only internal memory, or whether this disengagement extends to encoding strategies that are beneficial in the context of offloading. More broadly, the field should increase its focus on retrieval processes and the interaction between encoding and retrieval processes in cognitive offloading, particularly examining how different organizational strategies affect retrieval efficiency from external stores, and how individuals divide labor between encoding and retrieval.

## Conclusions

In our increasingly digital world, reliance on external information storage continues to grow. While cognitive offloading can benefit task performance, it has a detrimental effect on the internal trace of item-specific and relational aspects of the offloaded information. This study suggests that internal memory for relational information would provide limited support for the retrieval of information from external stores, particularly when information is distributed across multiple locations or organized in complex taxonomic systems. To optimize external store use, individuals should emphasize relational information in the structure and organization of external stores, developing strategies to enhance encoding and retrieval for externally stored information.

## Data Availability

The datasets generated during the current study are available in the Open Science Framework repository, https://osf.io/7mrv4/?view_only=b05f70afd1a545b3a055ed1848dbae7a. The materials used in the study appear in the Appendix.

## Appendix 1

See Tables

**Table 2:** Uncategorized word lists presented in blocks 1 and 2

List 1	List 2
Restaurant	Material
Opportunity	Tourist
Blue	Gold
Winter	Music
Meeting	Meaning
Message	Village
Morning	Broadcast
Street	Police
President	Experience
Idea	Respect
Discussion	Nature
Solution	Technology
Percentage	Answer
Hour	History
Grant	Painting
Shape	Screen
Movement	Version
Bank	Figure
Cinema	Conversation
City	Flag

2 and

**Table 3:** Categories and exemplars in each of the two categorized lists presented in block 3

Category	List A	List B
Fruit	Banana, apple, grape, watermelon, strawberry, pineapple	Orange, kiwi, plum, pear, grapefruit, peach
Clothing	Pants, sock, jumper, gloves, shoe, hat	Skirt, shirt, coat, dress, jacket, scarf
Furniture	Dresser, bed, ottoman, couch, shelf, armchair	Bookcase, drawer, stool, table, closet, chair
Kitchen	Pan, (bottle) opener*, mug, knife, pot, strainer	Ladle, plate, fork, spatula, bowl, spoon
Profession	Banker, teacher, fireman, doctor, engineer, secretary	Professor, policeman, carpenter, manager, psychologist, chef
Musical instruments	Electronic keyboard*, flute, cello, drum, piano, saxophone	Violin, harp, trumpet, harmonica, clarinet, guitar
Vehicles	Airplane, bicycle, bus, helicopter, train, jeep	Truck, motorcycle, boat, taxi, car, scooter
Sports	Football (soccer), swimming, running, tennis, basketball, wrestling	Judo, gymnastics, ski, volleyball, golf, boxing

Since there are no available normed taxonomies in Hebrew, we constructed the lists based on highly familiar categories and words. Importantly, the same lists were presented in the offloading and no-offloading conditions, and therefore, this aspect of the design would not undermine the interpretation of the results pertaining to any differences between the two groups

^*^One word in Hebrew

3.
